# Supplementary material for: Characterisation of the tilapia lake virus proteome and identification of an 11th protein, S9-F3
Source: Npj Viruses. 2026 Jan 9;4:2. doi: 10.1038/s44298-025-00167-0 (PMC12789600; doi:10.1038/s44298-025-00167-0)
Supplement: Supplementary file 1 — Supplementary figures [file 44298_2025_167_MOESM1_ESM.pdf]

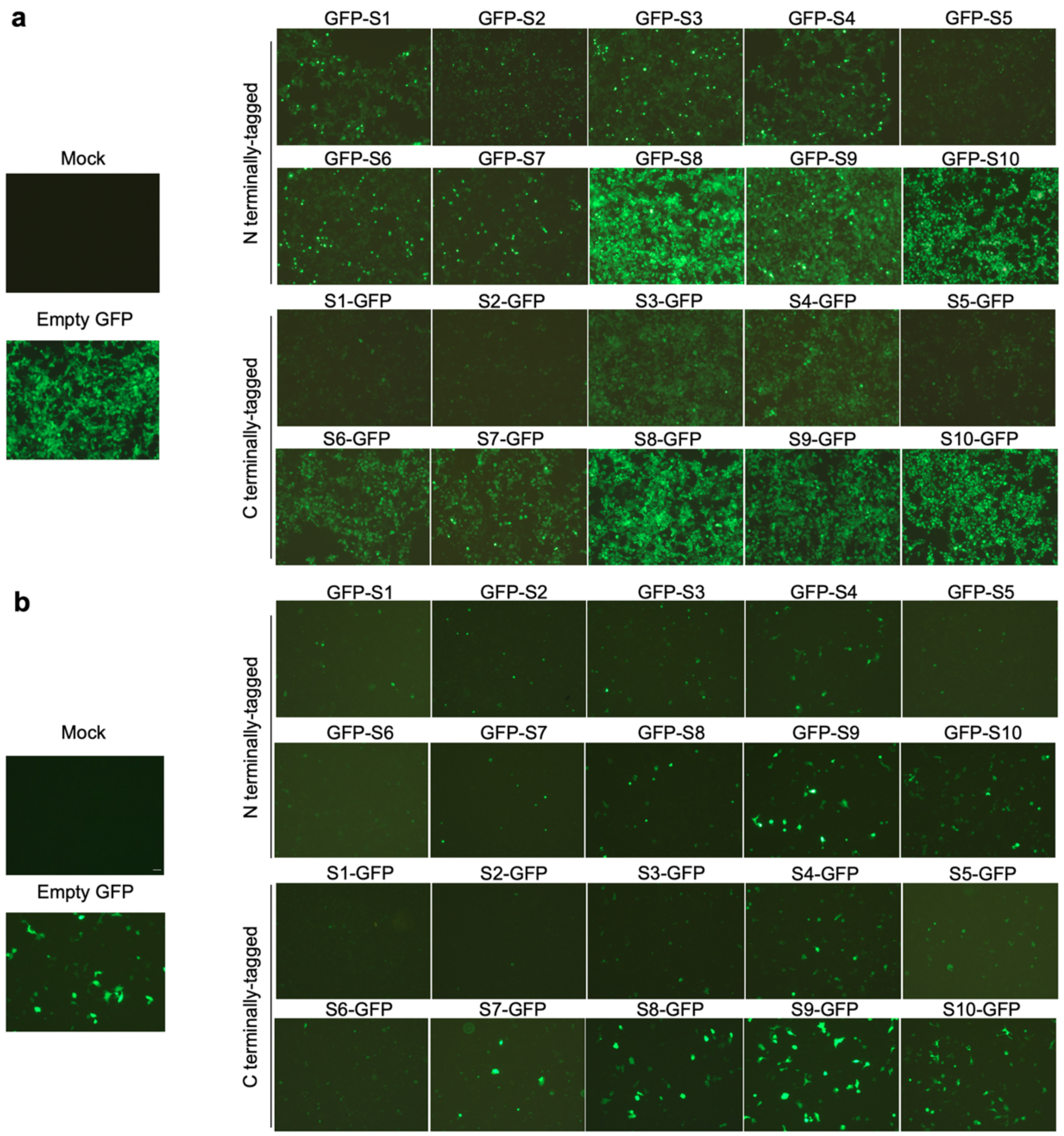

Supplementary Figure 1. Transfection efficiency of GFP-fusion S1-S10 proteins in mammalian and tilapia cells. 293T (a) and OmB (b) cells were transfected with indicated plasmids expressing TiLV proteins or empty GFP plasmid or mock controls. After 48 hours of transfection, live cells were visualised for positive GFP signal under a fluorescent microscope (Zeiss Axiovert 25) and images were acquired with a 10x objective.

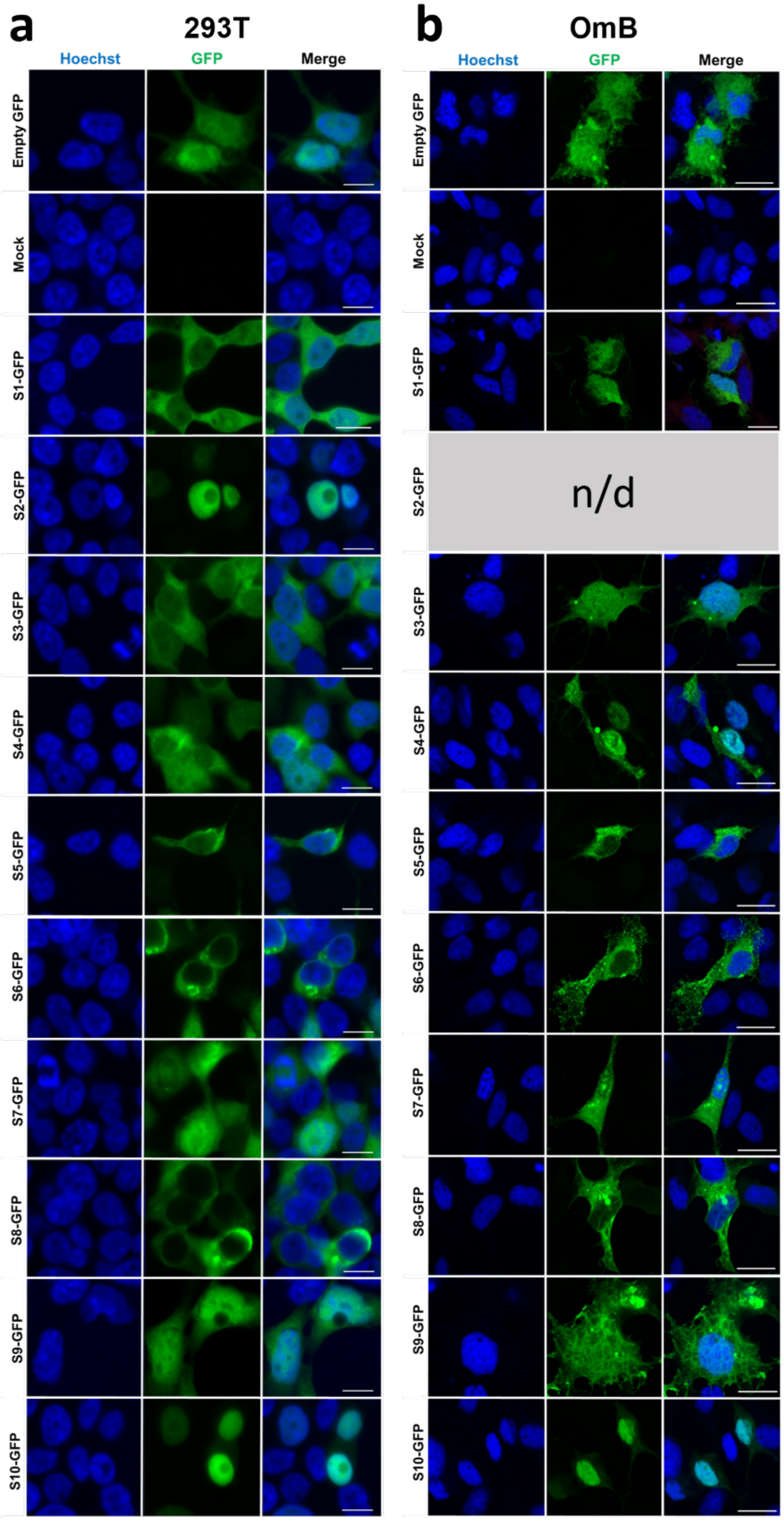
a
293T
Hoechst
GFP
Merge
Empty GFP
Mock
S1-GFP
S2-GFP
S3-GFP
S4-GFP
S5-GFP
S6-GFP
S7-GFP
S8-GFP
S9-GFP
S10-GFP
b
OmB
Hoechst
GFP
Merge
Empty GFP
Mock
S1-GFP
S2-GFP
n/d
S3-GFP
S4-GFP
S5-GFP
S6-GFP
S7-GFP
S8-GFP
S9-GFP
S10-GFP

Supplementary Figure 2. Subcellular localisation of GFP-tagged TiLV proteins in mammalian and tilapia cells. (a) 293T and (b) OmB cells were transfected with plasmids expressing C-terminal GFP fusion constructs of TiLV segments 1–10 or an empty GFP vector (pEGFP-C1), or were mock-transfected. 48 h post-transfection, cells were fixed and stained with Hoechst dye to visualise nuclei. GFP (green) and Hoechst (blue) fluorescence images were acquired using a Zeiss LSM710 microscope. Images are single optical slices, representative of three independent experiments, each performed with a single technical replicate. Scale bars: 10 μm, n/d: not determined because of poor expression.

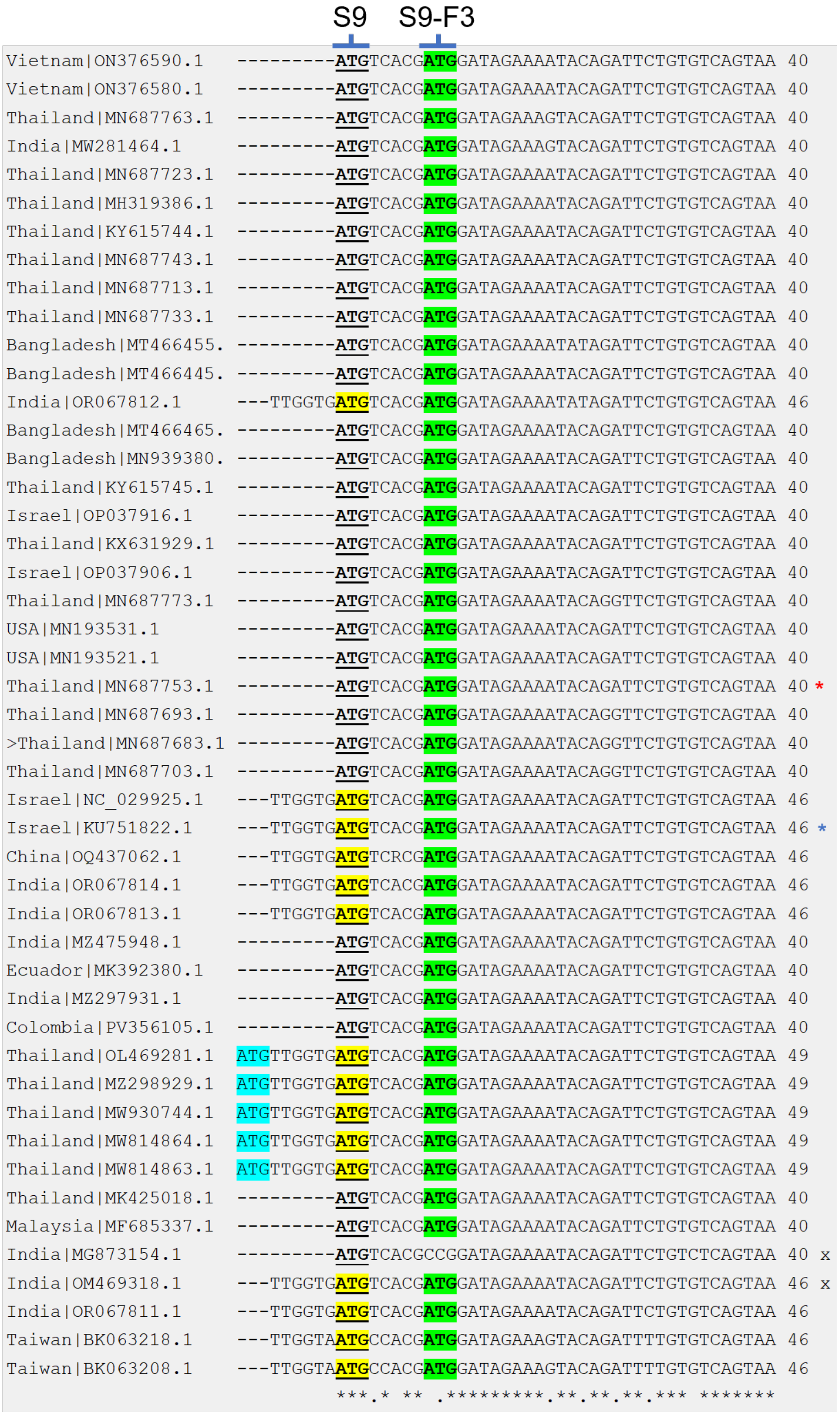

Supplementary Figure 3. Conservation analysis of TiLV S9 5′ sequences and Kozak consensus motifs. Forty-seven segment 9 sequences (plus sense, given as cDNA) were retrieved from the NCBI database and aligned using Clustal Omega, with visualization and annotation performed in Jalview. A partial alignment of the 5′ region is shown, with residue positions indicated at the end of each sequence. Conservation at each nucleotide position is marked by black asterisks below the alignment. The canonical start codons (ATG) for the

primary S9 ORF and the alternative S9-F3 ORF are highlighted, with yellow and green indicating intermediate and strong Kozak consensus strength, respectively. Additional upstream ATG codons (highlighted in blue) represent in-frame alternative start sites with, at most, intermediate Kozak consensus. Cross symbols denote two sequences excluded from the S9-F3 alignment due to the presence of a premature stop codon or the absence of an AUG initiation codon. Red and blue asterisks indicate sequences corresponding to the virus isolate and synthetic clones used in this study, respectively.

a

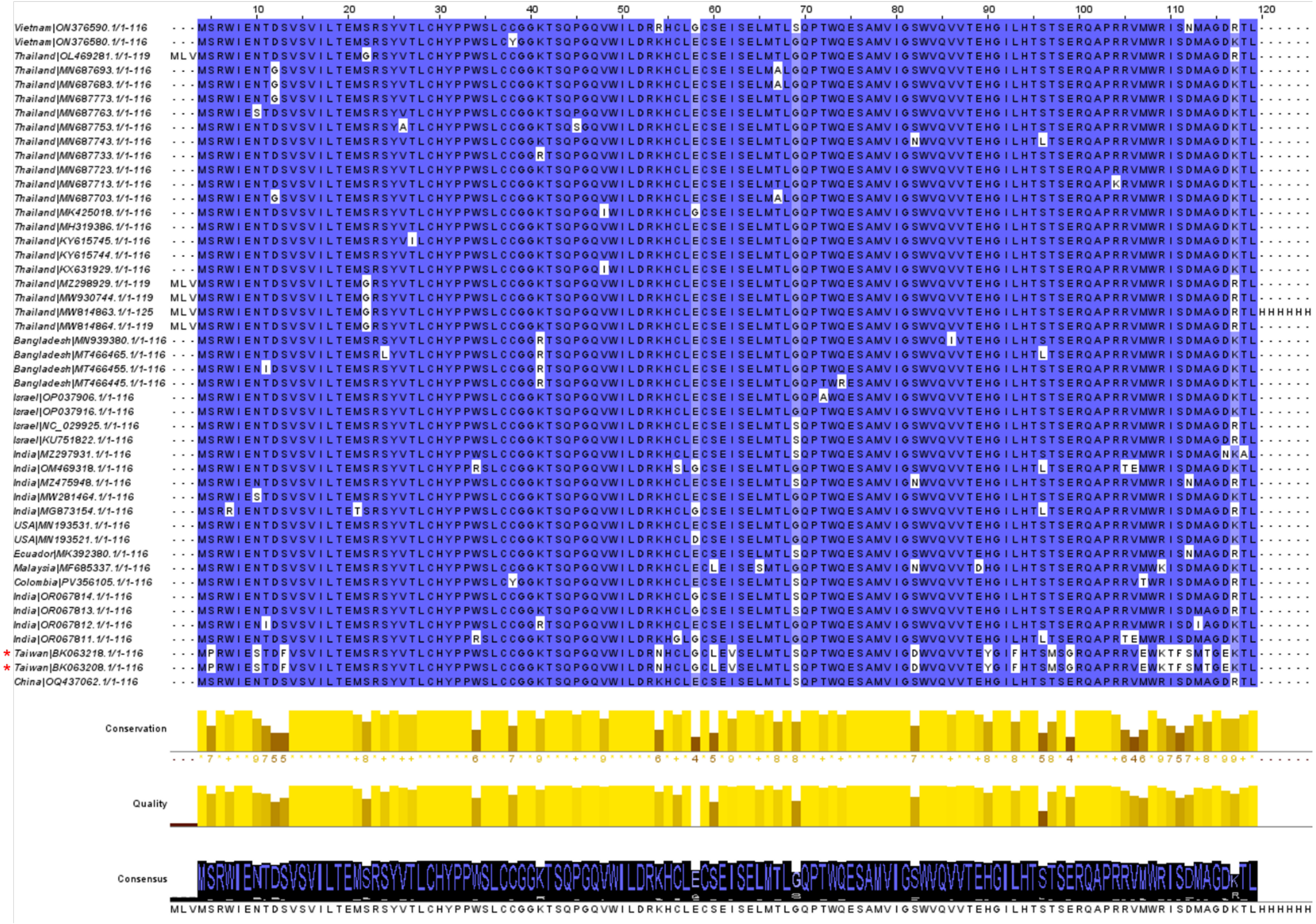
Vietnam|ON376590.1/1-116
Vietnam|ON376580.1/1-116
Thailand|OL469281.1/1-119
Thailand|MN687693.1/1-116
Thailand|MN687683.1/1-116
Thailand|MN687773.1/1-116
Thailand|MN687763.1/1-116
Thailand|MN687753.1/1-116
Thailand|MN687743.1/1-116
Thailand|MN687733.1/1-116
Thailand|MN687723.1/1-116
Thailand|MN687713.1/1-116
Thailand|MN687703.1/1-116
Thailand|MK425018.1/1-116
Thailand|MH319386.1/1-116
Thailand|KY615745.1/1-116
Thailand|KY615744.1/1-116
Thailand|KX631929.1/1-116
Thailand|MZ298929.1/1-119
Thailand|MW930744.1/1-119
Thailand|MW814863.1/1-125
Thailand|MW814864.1/1-119
Bangladesh|MN939380.1/1-116
Bangladesh|MT466465.1/1-116
Bangladesh|MT466455.1/1-116
Bangladesh|MT466445.1/1-116
Israel|OP037906.1/1-116
Israel|OP037916.1/1-116
Israel|NC_029925.1/1-116
Israel|KU751822.1/1-116
India|MZ297931.1/1-116
India|OM469318.1/1-116
India|MZ475948.1/1-116
India|MW281464.1/1-116
India|MG873154.1/1-116
USA|MN193531.1/1-116
USA|MN193521.1/1-116
Ecuador|MK392380.1/1-116
Malaysia|MF685337.1/1-116
Colombia|PV356105.1/1-116
India|OR067814.1/1-116
India|OR067813.1/1-116
India|OR067812.1/1-116
India|OR067811.1/1-116
* Taiwan|BK063218.1/1-116
* Taiwan|BK063208.1/1-116
China|OQ437062.1/1-116
Conservation
Quality
Consensus
MLVMSRWIENTDSVSVILTEMSRSYVTLCHYPPWSLCCGGKTSQPGQVWILDRKHCLECSEISELMTLGQPTWQESAMVIGSWVQVVTEHGILHTSTSERQAPRRVMWRISDMAGDKTLHHHHHH

b

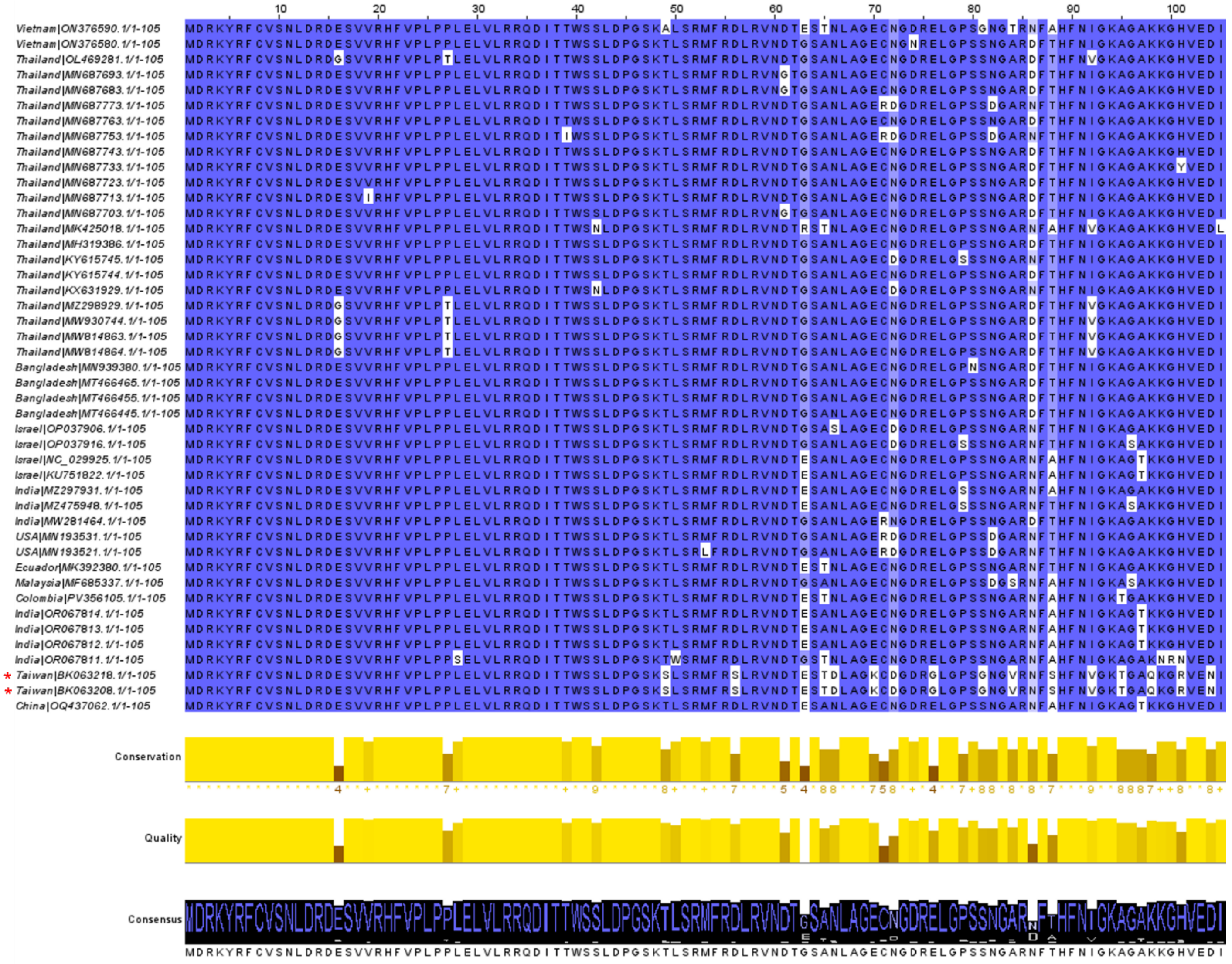
10
20
30
40
50
60
70
80
90
100
Vietnam|ON376590.1/1-105
Vietnam|ON376580.1/1-105
Thailand|OL469281.1/1-105
Thailand|MN687693.1/1-105
Thailand|MN687683.1/1-105
Thailand|MN687773.1/1-105
Thailand|MN687763.1/1-105
Thailand|MN687753.1/1-105
Thailand|MN687743.1/1-105
Thailand|MN687733.1/1-105
Thailand|MN687723.1/1-105
Thailand|MN687713.1/1-105
Thailand|MN687703.1/1-105
Thailand|MK425018.1/1-105
Thailand|MH319386.1/1-105
Thailand|KY615745.1/1-105
Thailand|KY615744.1/1-105
Thailand|KX631929.1/1-105
Thailand|MZ298929.1/1-105
Thailand|MW930744.1/1-105
Thailand|MW814863.1/1-105
Thailand|MW814864.1/1-105
Bangladesh|MN939380.1/1-105
Bangladesh|MT466465.1/1-105
Bangladesh|MT466455.1/1-105
Bangladesh|MT466445.1/1-105
Israel|OP037906.1/1-105
Israel|OP037916.1/1-105
Israel|NC_029925.1/1-105
Israel|KU751822.1/1-105
India|MZ297931.1/1-105
India|MZ475948.1/1-105
India|MW281464.1/1-105
USA|MN193531.1/1-105
USA|MN193521.1/1-105
Ecuador|MK392380.1/1-105
Malaysia|MF685337.1/1-105
Colombia|PV356105.1/1-105
India|OR067814.1/1-105
India|OR067813.1/1-105
India|OR067812.1/1-105
India|OR067811.1/1-105
* Taiwan|BK063218.1/1-105
* Taiwan|BK063208.1/1-105
China|OQ437062.1/1-105
Conservation
Quality
Consensus
MDRKYRFCVSNLDRDESVVRHFVPLPPLELVLRRQDITTWSSLDPGSKTLSRMFRDLRVNDTGSANLAGECNGDRELGPSSNGARNFTHFNIGKAGAKKGHVEDI

Supplementary Figure 4. Multiple sequence alignment of TiLV S9 and S9-F3 predicted polypeptide sequences. 47 complete coding sequences of S9 (a) and 45 of S9-F3 (b) were retrieved from the NCBI database and aligned via Clustal using Jalview software. Red asterisks indicate the guppy TiLV-like isolates from Taiwan. Aligned residues are color-coded based on conservation; a stronger intensity indicates a higher physicochemical property conservation score. Consensus represents the percentage of the most common residue per column; higher bars denote greater conservation. Alignment quality represents the likelihood of observing mutations in a specific position+, based on BLOSUM 62 scores; a higher quality score indicates fewer mutations. Conservation represents the amino acid sequence similarity at specific regions of the protein. Conserved columns are marked with an asterisk (*) with a score of 11, while columns with a single mutation, where all properties were conserved, are marked with a 'plus sign' (+) with a score of 10. Columns with less conservation, evidenced by multiple mutations, are assigned conservation scores less than 10.

Supplementary Data (see separate Excel files)

Supplementary Data 1. Summary of the major plus-strand ORFs in ten TiLV segments. Viral sequences were analysed for plus strand ORFs (minimum 25 codon length) using ORF Finder (NCBI). The position and length of the ORFs and the predicted protein sizes are given, along with Kozak consensus (strong, -3A/G and +4G; intermediate, -3A/G or + 4G; weak, neither -3A/G nor + 4G), with green letter indicates a codon corresponding to the consensus rule.

Supplementary Data 2. Computational analysis of predicted functional domains in TiLV proteins.

Supplementary Data 3. Six-frame translation of the TiLV genome. Six-frame translation software (Bioline) was used to identify all potential peptide sequences from the TiLV genome (Israel isolate, Til-4-2011, accession nos. KU751814–KU751823), with a minimum length of 9 amino acids. The predicted primary ORF sequence of each segment is highlighted in yellow. Plus (+) and minus (-) indicate translation from positive and negative strand, respectively. Numbers after the (+) and (-) represent the frame and number of the *in silico* translation results. For instance, >TiLVS1+2.7 represents the seventh translational product of segment 1, derived from frame 2 of positive strand.

Supplementary Data 4. TiLV peptides identified by mass spectrometry.

Supplementary Data 5. List of publicly available TiLV Segment 9 nucleotide sequences retrieved from the NCBI databases as of May 2025. The country of origin and accession numbers are shown. Sequences highlighted in red and marked with red asterisk indicate additional exclusions from the S9-F3 analysis due to mutations that disrupt the predicted S9-F3 ORF.

Supplementary Data 6. Oligonucleotides used for the construction of GFP-tagged TiLV plasmids.

Supplementary Data 7. Oligonucleotides used to introduce mutations into TiLV segment 9.
